# Prescriptions

**Published:** 1896-03

**Authors:** 


TO AID DIGESTION.

℞. Pepsini ............ ʒ i.

Acid. hydrochlor., dil... ʒ iv.

Glycerini ............... ʒ iv.

Strychninæ sulph........ gr. ss.

Aquæ chloroformi q.s.ad ℥ iii.

M. Sig.:—A teaspoonful in water after meals.

FOR DIARRHŒA.

℞. Tinct. Opii. deodorat.. ʒ v.

Spirits chloroformi..... ʒ ii.

Acid. sulphuric, dil.... ℥ i.

Vini pepsini........q. s. ℥ iv.

M. Sig.:—A teaspoonful in water after each stool.

FOR TAPE-WORM.

℞. Chloroformi.

Ext. aspidii fld......aa ʒ i.

Emul. olei ricini...... ℥ iii.

M. Sig.:—To be taken in the early morning; no food until thorough action of the bowels.

A GOOD DIURETIC.

℞. Potassii acetat......... ʒ v.

Inf. digital............. ℥ iii.

Liq. potassii citratis.... ℥ iii.

M. Sig.:—A tablespoonful every four hours in water.

Cases of anæmia with weak stomach can take the following "iron lemonade" with ease:

℞. Tr. ferri chloridi....... ʒi.

Acid. phosphor., dil... ʒii.

Syr. limonis............ ℥iss.

Aquæ.................... ℥ii.

M. Sig.:—One teaspoonful well diluted.

A GOOD TURPENTINE EMULSION.

℞. Olei terebinth...... ...ʒiii.

Olei gaultheriæ........gtt. vii.

Sacch. albæ............

Acaciæ, pulv.........aa q. s.

Glycerini................ ʒiv.

Aquæ..............q. s. ℥iii.

M. Sig.:—One to two teaspoonfuls every three hours.

FOR HEADACHE.

℞. Caffein, citrat ........ gr.xii.
Phenacetin.............gr. xx.
Sodii bromid............ ʒi.
Ft. Chart. No. 6.

M. Sig.:—One every two or three hours.

FOR DIPHTHERIA.

℞. Hydrogen dioxid........℥i.

Glycerin.................ʒii.

Aquæ, dist. .........℥ss.

M. Sig.:—Use as a spray or mop.

FOR ACNE.

℞. Sulphur, præcip........ ʒi.

Ung. aquæ rosæ,

Petrolat, moll........aa ʒiv.

M. Sig.:—Apply night and morning.

